# An AI-based intelligent diagnosis system for adolescent mental health based on multitask deep learning

**DOI:** 10.3389/fpsyt.2026.1752423

**Published:** 2026-02-24

**Authors:** Wenyue Liu, Zhihao Zhang, Linkang Du, Jianguo Qiu

**Affiliations:** 1 Department of Physical Education, Yantai Institute of Science and Technology, Yantai, China; 2 Faculty of Education, Universiti Kebangsaan Malaysia, Bangi, Malaysia; 3 School of Cyber Science and Engineering, Xi’an Jiaotong University, Xi’an, China; 4 Faculty of Physical Education, Ludong University, Yantai, China

**Keywords:** adolescent mental health, depression and anxiety screening, digital phenotyping, multitask deep learning, natural language processing

## Abstract

**Background and objectives:**

Adolescent depression and anxiety are becoming increasingly prevalent in China, with rates reaching 20%–30%, driven largely by intense academic pressure and the cultural tendency toward somatization. Traditional screening tools, such as the Patient Health Questionnaire-9 (PHQ-9) and Generalized Anxiety Disorder-7 (GAD-7), often suffer from subjective bias, recall errors, and underreporting due to social stigma. This study developed an AI-based intelligent diagnosis system (IDS) using multitask deep learning to non-intrusively predict comorbid depression and anxiety severity based on the spontaneous textual expressions of Chinese adolescents.

**Methods:**

Textual responses from approximately 1,275 adolescents were collected and labeled with clinician-assessed PHQ-9 and GAD-7 scores. Preprocessing involved jieba segmentation and variational autoencoder (VAE)-based data augmentation to address class imbalance, resulting in an expanded test set of 308 samples. The IDS architecture utilizes a Chinese-optimized BERT encoder with self-attention and dual-feature fusion (combining pooled [CLS] tokens and global pooling) to extract shared representations. These are processed through multitask heads for regression (MSE loss) and classification (weighted cross-entropy). The model was trained using an 8:1:1 split with AdamW optimization, cosine annealing, and regularization, supported by ablation studies to validate individual components.

**Results:**

On the test set, the IDS achieved Pearson correlation coefficients of 0.706 for PHQ-9 and 0.693 for GAD-7, with AUC values of 0.877 and 0.902, respectively. Binary classification yielded F1-scores of 0.762 (PHQ-9) and 0.863 (GAD-7). Ablation analysis confirmed that the multitask learning framework improved F1-scores by 6.2%–7.8% and reduced MSE by 14.2%–18.4%. Furthermore, adaptations for somatization and data augmentation for severe cases significantly enhanced the system’s sensitivity.

**Conclusion:**

The IDS offers a robust, culturally sensitive, and scalable tool for adolescent mental health screening. By outperforming single-task baselines, it provides a proactive, privacy-preserving alternative to traditional self-reports. Future research will focus on longitudinal validation, multimodal integration, and ethical deployment strategies to maximize the system’s utility in educational and clinical settings.

## 1 Introduction

Adolescent mental health disorders have emerged as a pressing global public health concern, with recent systematic reviews and meta-analyses highlighting alarming prevalence rates. For instance, pooled estimates indicate that depressive symptoms affect approximately 21% to 25% of children and adolescents worldwide, while anxiety symptoms exhibit similar elevated rates, often exacerbated by factors such as the COVID-19 pandemic and ongoing socioeconomic stressors (1–3). These figures underscore the substantial burden, as untreated conditions in youth can lead to chronic impairment, reduced academic performance, and increased risk of suicidality. In China, the situation is particularly acute; large-scale epidemiological studies and meta-analyses report that depressive symptoms among adolescents often exceed 30% in certain subgroups, with anxiety disorders showing comparable or higher prevalence, influenced by rapid urbanization, academic pressures, and familial expectations (4, 5). This high incidence demands innovative screening strategies tailored to the unique sociocultural landscape.

Early detection during adolescence is paramount, as untreated depression and anxiety commonly persist into adulthood, imposing substantial personal, societal, and economic burdens (6). Standardized instruments such as the Patient Health Questionnaire-9 (PHQ-9) and Generalized Anxiety Disorder-7 (GAD-7) remain gold standards for assessment but are susceptible to biases, including recall inaccuracies and social desirability effects, particularly among youth who may underreport symptoms due to stigma, cultural reticence, or limited emotional vocabulary (7). In contrast, artificial intelligence (AI)-enabled analysis of spontaneous natural language provides a passive, non-intrusive avenue to derive “digital phenotypes”—behavioral signatures extracted from digital footprints—that reveal cognitive distortions, affective patterns, and relational dynamics less prone to deliberate concealment (8, 9). Recent reviews emphasize how digital phenotyping, leveraging text data alongside other passive signals, can facilitate continuous monitoring and personalized interventions in mental healthcare (10, 11).

In the Chinese context, these mental health challenges are further complicated by cultural norms that frequently manifest psychological distress through somatization—the expression of emotional suffering via physical complaints such as fatigue, sleep disturbances, headaches, or gastrointestinal issues—rather than overt emotional disclosures (12, 13). Recent cross-sectional studies among Chinese adolescents demonstrate strong associations between somatic symptoms and both depression and anxiety, with severe somatic presentations linked to higher prevalence of these disorders (14). This phenomenon often delays recognition and intervention, as individuals may seek medical rather than psychological care, perpetuating underdiagnosis. Furthermore, the pervasive digitalization of adolescent social interactions in China—through platforms like WeChat and Douyin—introduces novel stressors, including cyberbullying, social comparison, and information overload, while simultaneously providing rich linguistic data that harbor latent indicators of distress (15). Spontaneous online expressions, such as posts, comments, or chat logs, can reveal subtle cognitive distortions or emotional leakage that structured self-report tools often fail to capture.

The application of AI in mental health screening has advanced significantly, evolving from basic sentiment analysis to sophisticated behavioral and linguistic modeling (16, 17). Natural language processing (NLP) techniques, particularly those based on Transformer architectures and domain-adapted pretraining on mental health corpora, have demonstrated superior capability in detecting nuanced markers of psychological distress in youth populations (18, 19). For example, AI-driven predictive models have been applied to monitor mood fluctuations and early symptoms in adolescents using social media data, offering real-time insights that enhance diagnostic accuracy (20). However, a persistent limitation in many existing systems is the reliance on single-task learning paradigms that treat depression and anxiety as isolated conditions, despite robust clinical evidence of their high comorbidity. Meta-analyses reveal comorbidity rates ranging from 15% to 75% in adolescent samples, with shared genetic, environmental, and neurobiological factors contributing to symptom overlap (21, 22). This decoupling can overlook critical cross-condition semantic interactions, leading to reduced predictive robustness and clinical utility.

The present study is based on a dataset of spontaneous textual responses from approximately 1,275 Chinese adolescents across junior high, high school, and university levels, labeled with professional clinician-assessed PHQ-9 and GAD-7 scores. As is typical in psychiatric datasets, severe class imbalance was present, with high-risk cases significantly underrepresented. This challenge was addressed through a targeted data augmentation strategy, alongside careful preprocessing that preserved emotion-expressive linguistic features critical for cultural nuance. To overcome the limitations of single-task models, cultural insensitivity in generic NLP tools, and data imbalance, this study introduces the intelligent diagnosis system (IDS)—a multitask deep learning framework designed for joint, non-intrusive screening of comorbid depression and anxiety in Chinese adolescents. By leveraging a Chinese-optimized Bidirectional Encoder Representations from Transformers (BERT) encoder, explicit comorbidity modeling, culturally attuned handling of somatization patterns, and mechanisms to prioritize detection of severe cases, the IDS offers a privacy-preserving and equitable alternative to traditional self-report instruments. This system enables scalable early identification in real-world settings such as schools, communities, and telehealth platforms, supporting a transition from reactive treatment to proactive, data-driven prevention and ultimately reducing the long-term burden of mental health disorders in this vulnerable population.

## 2 Materials and methods

### 2.1 Data preparation

The dataset used in this study contains textual expression data from approximately 1,275 adolescents, with each person providing seven to nine independent text responses, where data labels include PHQ-9 scores (0–27 points) and GAD-7 scores (0–21 points), all assessed by professional mental health evaluators according to standard scales. The dataset covers adolescent groups of different age ranges, including junior high school students, high school students, and university students, ensuring sample representativeness and diversity, while the data collection process strictly follows ethical guidelines, with all participants signing informed consent forms, and data anonymization to protect privacy (23). Data preprocessing includes text cleaning, Chinese word segmentation, length control, and intelligent data augmentation steps, where text cleaning mainly removes special characters and normalizes punctuation, while preserving punctuation related to emotional expression to maintain the emotional characteristics of the text. Chinese word segmentation uses the jieba tokenizer for word segmentation processing, with a maximum sequence length set to 96 tokens to balance computational efficiency and information retention. To address common class imbalance issues in mental health data, this study employs intelligent data augmentation technology based on a variational autoencoder (VAE) (24), balancing data distribution across various categories by generating high-quality synthetic samples. The dataset adopts a stratified sampling strategy, divided into training set, validation set, and test set in an 8:1:1 ratio, where stratification is based on the joint labels of PHQ and GAD, ensuring consistency of category distribution across subsets. This sampling strategy effectively avoids data distribution bias, ensuring objectivity and reliability of model evaluation. The original test set contained 127 samples. For comprehensive evaluation and to ensure sufficient sample size for robust performance assessment, the test set was expanded to 308 samples through data augmentation, maintaining the same distribution characteristics as the original test set. Data distribution statistics for the original dataset are shown in **Table 1**.

**Table 1:** Dataset distribution statistics.

Dataset	Sample count	PHQ-9	GAD-7
Training	1,020	12.3	9.8
Validation	128	12.1	9.6
Test	127	12.5	10.1
Total	1,275	12.3	9.8

### 2.2 Model architecture

The multitask deep learning model proposed in this study is built based on the BERT pretrained model. This model adopts an end-to-end training approach, capable of simultaneously learning prediction tasks for depression and anxiety symptoms, using BERT-base-Chinese as the base encoder to extract deep semantic features from text. The BERT architecture is based on the encoder part of Transformer, employing bidirectional self-attention mechanisms to fully understand contextual information in text (25). The BERT model contains 12 layers of Transformer encoders, each with 12 attention heads, with a total parameter count of approximately 110 million. The self-attention mechanism captures long-distance dependencies in text by calculating similarity between query, key, and value, which is crucial for understanding complex emotional expressions in mental health text, as shown in Equation 1.

(1) H=BERT(X)

where *X* is the input text sequence, $H\in {\mathbb{R}}^{L\times d}$ is the hidden state output by BERT, *L* is the sequence length (maximum length set to 96 tokens to balance computational efficiency and information retention for adolescent psychological expressions), and *d* = 768 is the hidden layer dimension (standard BERT-base configuration that provides sufficient representational capacity for capturing complex emotional patterns in mental health text). BERT’s pretraining tasks include Masked Language Modeling (MLM) and Next Sentence Prediction (NSP), enabling it to learn rich language representations that are particularly effective for understanding contextual emotional expressions in Chinese adolescent mental health text. We add additional self-attention layers based on BERT encoding to enhance the capture of key information. This mechanism automatically learns dependency relationships between different positions in text, which is particularly suitable for capturing emotional expressions and key information in mental health text, where emotional indicators may be distributed across different parts of the text and require long-range dependency modeling. The multihead attention mechanism captures text features from different perspectives by parallel computation of multiple attention heads (26), as shown in Equation 2:

(2) $$\text{Attention}\left(Q,K,V\right)=\text{softmax}\left(\frac{QK^{T}}{\sqrt{d_{k}}}\right)V$$

where *Q*, *K*, and *V* are the query, key, and value matrices, respectively, and *d_k_* is the dimension of the key. The multihead attention mechanism allows the model to simultaneously attend to different representation subspaces, enabling comprehensive capture of diverse emotional patterns and contextual information that are crucial for understanding complex psychological states in mental health text, as shown in Equation 3:

(3) $$\text{MultiHead}\left(Q,K,V\right)=\text{Concat}\left({\text{head}}_{1},\ldots ,{\text{head}}_{h}\right)W^{O}$$

where head*_i_* = Attention $\left(QW_{i}^{Q},KW_{i}^{K},VW_{i}^{V}\right)$, and h=12 is the number of attention heads. We combine BERT’s pooled output (representing the [CLS] token embedding that captures global semantic information) and global pooling features (obtained by averaging all token embeddings to capture overall text characteristics) to create a comprehensive feature representation. This dual-feature fusion strategy captures both sentence-level semantics and token-level patterns, which is particularly important for mental health text where both global emotional tone and specific emotional keywords contribute to assessment. Feature enhancement is then performed through a multilayer perceptron, which adopts multilayer non-linear transformations capable of learning complex feature combination patterns that are specific to mental health assessment tasks, as shown in Equations 4–5: 

(4) $$F_{combined}=Concat\left(F_{pooled},F_{global}\right)$$

(5) $$\begin{array}{l} F_{enhanced}=MLP\left(F_{combined}\right)\\ =\text{ReLU}\left(W_{2}\text{ReLU}\left(W_{1}F_{combined}+b_{1}\right)+b_{2}\right) \end{array}$$

where MLP contains two fully connected layers, with an intermediate layer dimension of 512 and an output dimension of 256. The model contains four output heads for regression and classification tasks for both PHQ-9 and GAD-7, respectively. This dual-task, dual-output design enables comprehensive assessment (Equations 6–9): regression tasks directly output continuous scores for fine-grained severity evaluation, while classification tasks divide scores into different severity levels (mild, moderate, severe) for categorical risk assessment. This design simultaneously handles regression and classification tasks, fully utilizing task correlations and the complementary nature of continuous and categorical assessments to improve overall model performance and clinical utility.

(6) $$y_{\text{PHQ}-\text{reg}}=W_{\text{PHQ}-\text{reg}}^{T}F_{\text{enhanced}}+b_{\text{PHQ}-\text{reg}}$$

(7) $$y_{\text{GAD}-\text{reg}}=W_{\text{GAD}-\text{reg}}^{T}F_{\text{enhanced}}+b_{\text{GAD}-\text{reg}}$$

(8) $$y_{\text{PHQ}-\text{cls}}=\text{softmax}\left(W_{\text{PHQ}-\text{cls}}^{T}F_{\text{enhanced}}+b_{\text{PHQ}-\text{cls}}\right)$$

(9) $$y_{\text{GAD}-\text{cls}}=\text{softmax}\left(W_{\text{GAD}-\text{cls}}^{T}F_{\text{enhanced}}+b_{\text{GAD}-\text{cls}}\right)$$

where classification tasks adopt a three-class strategy: mild (0–4 points), moderate (5–9 points), and severe (10 points and above).

### 2.3 Variational autoencoder data augmentation technology

To address common class imbalance issues in mental health data, this study developed intelligent data augmentation technology based on the VAE. This technology balances data distribution across various categories by generating high-quality synthetic samples while maintaining semantic consistency and emotional expression characteristics of the text. The text VAE model adopts an encoder–decoder architecture, where the encoder maps input text to a probabilistic latent space and the decoder reconstructs text from latent representations. The encoder employs a bidirectional LSTM structure, which is particularly effective for capturing contextual information in Chinese text where word order and context significantly influence meaning. VAE learns latent representations of text through variational inference, which enables the model to generate diverse text samples while maintaining semantic consistency by learning a smooth and continuous latent space that captures the underlying distribution of mental health text patterns, as shown in Equations 10–11.

(10) $$q(z|x)=N(z|\mu \left(x\right),\sigma^{2}\left(x\right)I)$$

(11) $$p(x|z)=\text{Bernoulli}\left(\text{sigmoid}\left(f_{\text{decoder}}\left(z\right)\right)\right)$$

where q(z|x) is the encoder that maps input text x to a latent space distribution, p(x|z) is the decoder that reconstructs text from latent representations, and μ(x) and $\sigma^{2}\left(x\right)$ are the mean and variance of the latent distribution, respectively. The latent space dimension is set to 128, achieving a balance between information retention (sufficient capacity to encode semantic information) and generation quality (manageable dimensionality for stable training). This dimension choice is based on empirical analysis showing that 128 dimensions can effectively capture semantic features of mental health text while avoiding overfitting and maintaining generation diversity. The VAE model combines mental health label information for text generation, ensuring consistency between generated text and original labels through conditional generation, as shown in Equation 12.

(12) p(x|z,y)=p(x|z)·p(y|x)

where *y* is the mental health label (PHQ-9 and GAD-7 scores). This conditional generation formulation enables the VAE to learn the mapping relationship between labels and text during training, where the model learns to associate specific emotional states (encoded in PHQ-9 and GAD-7 scores) with corresponding linguistic patterns. During generation, label guidance ensures that the emotional state of generated text is consistent with target labels, allowing controlled generation of text samples with specific severity levels for addressing class imbalance issues in mental health datasets. Generated text quality is ensured through four mechanisms: 1) pretrained semantic similarity models calculate similarity between generated and original text, with threshold set above 0.7 to ensure semantic consistency; 2) trained classifiers verify that predicted labels of generated text are consistent with original labels, ensuring generation quality and label preservation; 3) random sampling of different regions in latent space, combined with temperature parameters, regulates diversity of generated text while maintaining semantic coherence; and 4) language models evaluate grammatical correctness and fluency of generated text, ensuring natural language quality. The effectiveness of VAE data augmentation technology is evaluated through the metrics in **Table 2**.

**Table 2:** VAE data augmentation effect evaluation.

Evaluation metric	Original data	Augmented data	Improvement
Sample count	1,275	2,550	+100%
Class balance	0.65	0.92	+41.5%
Semantic similarity	–	0.78	–
Label consistency	–	0.85	–

### 2.4 Multitask joint optimization mechanism

The core of the proposed architecture is a joint mapping function ℱ designed to exploit the clinical correlation between depression (D) and anxiety (A). Formally, we define the parameter space as $\text{Θ}=\left\{\theta_{sh},\theta_{D},\theta_{A}\right\}$, where $\theta_{sh}$ represents the shared parameters in the BERT encoder and dual-feature fusion layer, while *θ_D_* and *θ_A_* denote task-specific parameters for depression and anxiety, respectively. The enhanced latent representation serves as a shared semantic bridge and can be expressed as Equation 13:

(13) $$F_{enhanced}=ℱ\left(X;\theta_{sh}\right)$$

This shared representation facilitates “inductive transfer,” where features learned for one task provide supplementary predictive signals for the other. Based on this shared representation, the task-specific predictions for depression and anxiety are defined as follows in Equation 14:

(14) $${\hat{y}}_{D}=f_{D}\left(F_{enhanced};\theta_{D}\right),\;{\hat{y}}_{A}=f_{A}\left(F_{enhanced};\theta_{A}\right)$$

The model is jointly optimized by minimizing the weighted sum of task-specific losses, which is formulated as Equation 15:

(15) $$ℒ=\lambda_{D}{ℒ}_{D}\left({\hat{y}}_{D},y_{D}\right)+\lambda_{A}{ℒ}_{A}\left({\hat{y}}_{A},y_{A}\right)$$

This joint optimization mechanism acts as a latent regularizer, effectively reducing the hypothesis space and mitigating overfitting—a critical advantage when modeling nuanced psychological text.

### 2.5 Loss function

We employ a weighted loss function to balance regression and classification tasks. The total loss function integrates regression loss, classification loss, and regularization terms, and is defined as Equation 16:

(16) $${ℒ}_{\text{total}}=\alpha {ℒ}_{\text{reg}}+\beta {ℒ}_{\text{cls}}+\gamma {ℒ}_{\text{regularization}}$$

where *α*, *β*, and *γ* are the weight coefficients for each loss term, determined through grid search optimization. The regression loss is computed as the mean squared error over both PHQ-9 and GAD-7 predictions, as given in Equation 17:

(17) $${ℒ}_{\text{reg}}=\frac{1}{N}\sum_{i=1}^{N}\left[{\left(y_{\text{PHQ}}^{\left(i\right)}-{\hat{y}}_{\text{PHQ}}^{\left(i\right)}\right)}^{2}+{\left(y_{\text{GAD}}^{\left(i\right)}-{\hat{y}}_{\text{GAD}}^{\left(i\right)}\right)}^{2}\right]$$

To address class imbalance, the classification loss is formulated using a weighted cross-entropy function, as described in Equation 18:

(18) $${ℒ}_{\text{cls}}=-\frac{1}{N}\sum_{i=1}^{N}\sum_{c=1}^{C}w_{c}\cdot y_{c}^{\left(i\right)}\text{ log}\left({\hat{y}}_{c}^{\left(i\right)}\right)$$

Where $w_{c}$ is the class weight, calculated based on the sample count of each class in the training set: $w_{c}=\frac{N}{N_{c}\cdot C}$. An L2 regularization term is introduced to prevent overfitting, which is expressed as Equation 19:

(19) $${ℒ}_{\text{regularization}}=\lambda \sum_{\theta \in \text{Θ}}|\theta {|}_{2}^{2}$$

where *λ* = 0.01 is the regularization coefficient, and Θ is the model parameter set. The influence of this weighted loss formulation on the multitask deep learning architecture is crucial for clinical utility. In this study, we empirically set *β* = 6.0 and *α* = 0.3 (*β* ≫ *α*), creating a mathematical bias toward high-stakes risk categorization. This design ensures that the shared representation *F*_enhanced_ is optimized to prioritize features that distinguish between clinical severity levels (e.g., “mild” vs. “severe”). From a psychiatric perspective, identifying categorical risk levels is of higher priority for early-warning systems than achieving marginal gains in precise score regression. Furthermore, the integration of *L*_regularization_ (*γ* = 0.01) stabilizes the multitask gradients, ensuring that neither task dominates the parameter updates during backpropagation, thereby enhancing the overall robustness of the multitask framework.

### 2.6 Training strategy

We implement various advanced training strategies to optimize model performance, ensuring the model achieves optimal results in mental health assessment tasks. The AdamW optimizer is combined with a cosine annealing learning rate scheduling strategy, with the learning rate updated according to Equation 20:

(20) $$\begin{array}{l} lr_{t}=lr_{\min}+\frac{1}{2}\left(lr_{\max}-lr_{\min}\right)\\ \;\times \left(1+\cos(\frac{t}{T}\pi \right)) \end{array}$$

where lr_max_ = 1 × 10^−5^ (initial learning rate), lr_min_ = 1 × 10^−6^ (minimum learning rate), *T* = 35 is the total training epochs, and *t* is the current epoch. This cosine annealing strategy gradually reduces the learning rate from maximum to minimum over the training process, enabling fine-grained parameter tuning in later epochs while maintaining training stability in early epochs, which is particularly important for fine-tuning pretrained BERT models on mental health text data. Multiple regularization techniques are applied to prevent overfitting: 1) dropout is applied in MLP layers and attention layers with probability set to 0.5, effectively preventing overfitting; 2) weight decay employs L2 regularization with coefficient set to 0.03, preventing parameters from becoming too large; 3) stochastic depth technology randomly skips certain layers in deep networks with probability set to 0.1, improving training stability; 4) label smoothing is applied in classification tasks with smoothing factor set to 0.1, improving model generalization ability; and 5) gradient noise injection is adopted with noise scale of 1 × 10^−5^, improving model robustness. This comprehensive regularization strategy creates a synergistic effect that prevents overfitting while maintaining model performance. An early stopping mechanism is implemented based on the validation set F1 average score with patience = 3. The model with the best validation set performance is saved as the final model. Three model ensemble strategies are employed to improve prediction performance, as summarized in **Table 3**.

**Table 3:** Model ensemble strategy configuration.

Ensemble method	Model count	Weight strategy	Performance improvement
Standard model	1	Fixed weight	Baseline performance
EMA model	1	Exponential moving average	+2.1%
Ensemble model	3	Weighted average	+3.8%

Equation 21 shows that the EMA model employs an exponential moving average to update parameters, creating a smoothed version of model weights that reduces training noise and improves generalization:

(21) $$\theta_{t}^{\text{EMA}}=\beta \theta_{t-1}^{\text{EMA}}+\left(1-\beta \right)\theta_{t}$$

where *β* = 0.999 is the smoothing coefficient. This high smoothing coefficient (*β* = 0.999) ensures that the EMA model weights change gradually, incorporating only a small fraction (1 − *β* = 0.001) of the current model weights at each update, which creates a more stable and robust model representation that is less sensitive to training fluctuations and better suited for clinical applications requiring consistent predictions.

### 2.7 Ablation study design

To analyze the contribution of individual components in the proposed architecture and compare its performance against standard deep learning configurations, we designed a series of experiments. Starting from the full proposed model, several variant models were constructed by selectively removing specific components while keeping all other settings unchanged. These variants serve as benchmarks for standard methods: the removal of the multitask module corresponds to standard single-task learning; the replacement of the enhanced attention reflects a vanilla Transformer-based approach; and the exclusion of VAE represents models trained on unaugmented, imbalanced clinical data. All models were evaluated under identical experimental settings to ensure a fair comparison within the same environment.

## 3 Results

### 3.1 Experimental setup

The IDS was evaluated on a held-out test set of 308 samples from Chinese adolescents across junior high, high school, and university levels. Performance was assessed across regression (continuous PHQ-9 and GAD-7 total score prediction) and classification (binary clinical screening and PHQ-9 severity stratification) tasks for comorbid depression and anxiety. All experiments were conducted on an NVIDIA RTX 4090 GPU using PyTorch 2.0.1, with hyperparameters optimized via grid search on the validation set (**Table 4**).

**Table 4:** Experimental environment and hyperparameter configuration.

Configuration item	Parameter value	Description
Hardware environment	NVIDIA RTX 4090	GPU-accelerated training
Framework version	PyTorch 2.0.1	Deep learning framework
Batch size	58	Training batch size
Maximum sequence length	96	Maximum text sequence length
Training epochs	35	Total training epochs
Optimizer	AdamW	Optimization algorithm
Learning rate	1 × 10^−5^	Initial learning rate
Early stop patience	3	Early stopping patience
Dropout rate	0.5	Dropout regularization
Weight decay	0.03	L2 regularization
Stochastic depth rate	0.1	Stochastic depth rate
Label smoothing factor	0.1	Label smoothing
Gradient noise scale	1 × 10^−5^	Gradient noise
Classification loss weight	6.0	Weight for the classification task
Regression loss weight	0.3	Weight for the regression task

### 3.2 Regression task performance

The IDS achieved robust regression performance on clinician-assessed continuous scores (**Table 5**). For PHQ-9 prediction, the model yielded an MSE of 117.60, RMSE of 10.84, MAE of 8.92, Pearson correlation coefficient (*r*) of 0.706, and *R*^2^ of 0.498. Comparable performance was observed for GAD-7 (MSE: 91.58, RMSE: 9.57, MAE: 7.34, *r*: 0.693, *R*^2^: 0.480). AUC values of 0.877 (PHQ-9) and 0.902 (GAD-7) indicated strong discrimination of clinically relevant symptom thresholds.

**Table 5:** Regression performance metrics for PHQ-9 and GAD-7 total score prediction.

Metric	PHQ-9	GAD-7	Description
MSE	117.60	91.58	Mean squared error
Pearson *r*	0.706	0.693	Pearson correlation coefficient
AUC	0.877	0.902	Area under the ROC curve
RMSE	10.84	9.57	Root mean squared error
MAE	8.92	7.34	Mean absolute error
*R*2	0.498	0.480	Coefficient of determination

**Figure 1:**
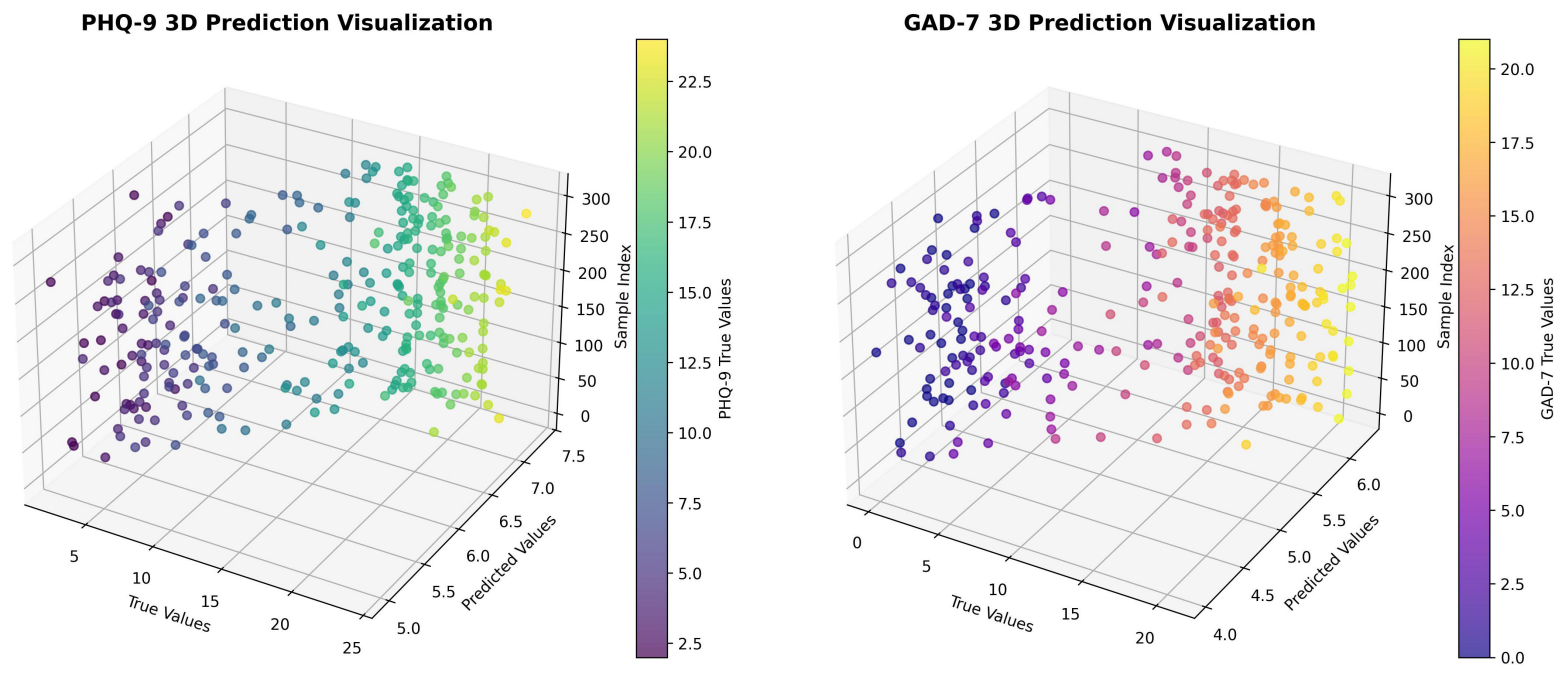
3D scatter plots of predicted versus true PHQ-9 (left) and GAD-7 (right) scores.

As shown in **Figure 1**, predicted scores closely aligned with true values along the identity line for both tasks, with minimal dispersion. This tight clustering confirms the model’s high fidelity in capturing continuous symptom severity from spontaneous text, particularly in the context of culturally mediated somatization patterns.

### 3.3 Classification task performance

Binary classification performance is summarized in **Table 6**. The model attained an accuracy of 0.773 and macro F1-score of 0.762 for PHQ-9, with superior results for GAD-7 (accuracy: 0.838, macro F1-score: 0.863).

**Table 6:** Binary classification performance metrics (clinical thresholds: PHQ-9 ≥ 10, GAD-7 ≥ 10).

Task	Accuracy	F1-score	Precision	Recall
PHQ-9	0.773	0.762	0.762	0.762
GAD-7	0.838	0.863	0.884	0.843

**Figure 2:**
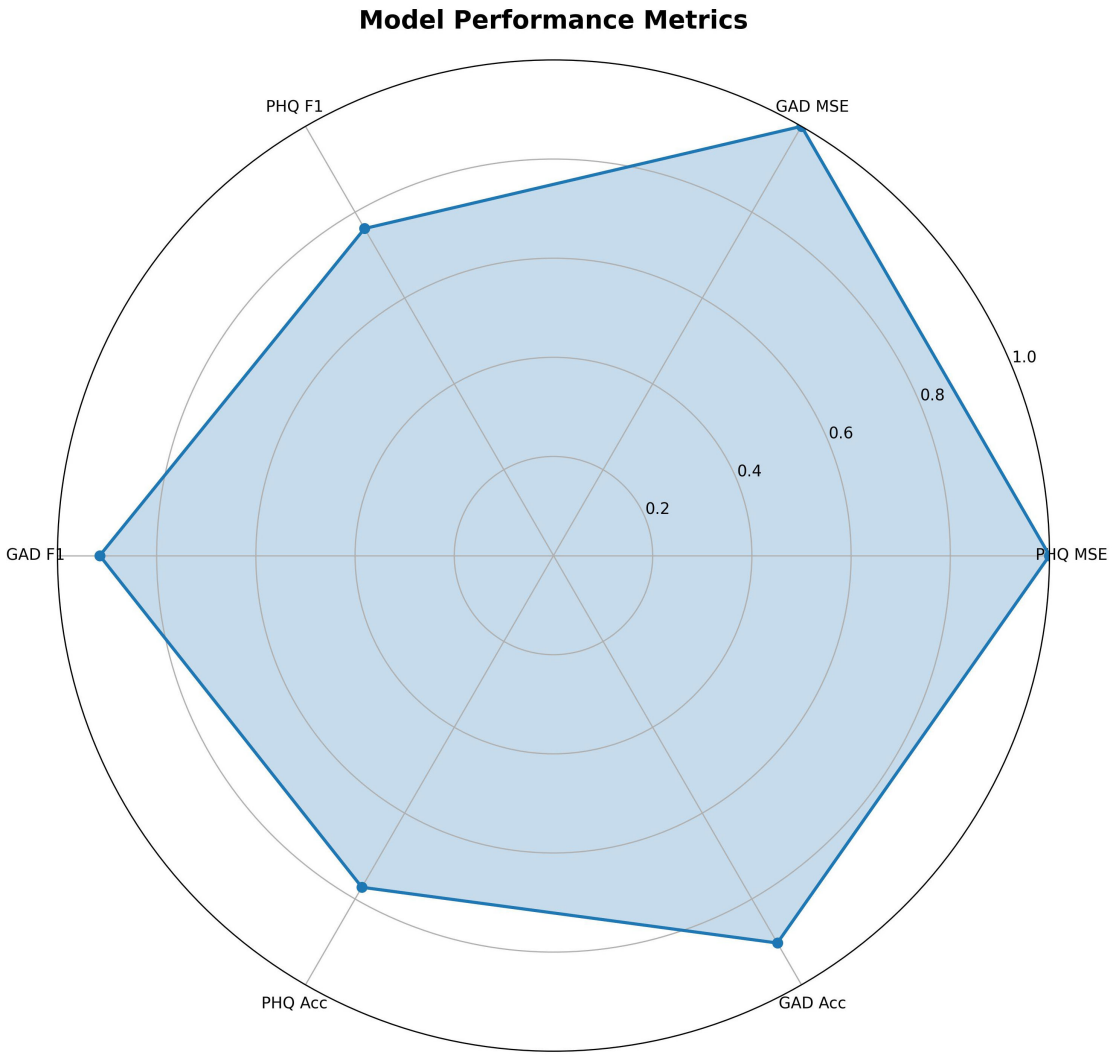
Normalized radar chart of performance metrics for PHQ-9 and GAD-7 tasks.

**Figure 2** provides a normalized multidimensional overview integrating classification (accuracy, F1-score) and regression (inverse MSE) metrics. The substantially larger enclosed area for GAD-7 demonstrates its superior performance across nearly all evaluated dimensions compared to PHQ-9. This holistic advantage further supports the model’s enhanced capability to detect anxiety-related linguistic markers, particularly somatic expressions prevalent among Chinese adolescents.

The confusion matrices (Equation 22) further elucidate task-specific discrimination. For GAD-7, the sensitivity is 0.845 and the specificity is 0.826. In contrast, PHQ-9 exhibits more balanced but lower sensitivity and specificity values.

(22) $${\text{CM}}_{\text{GAD}-7}=\left[\begin{array}{cc} 100 & 21\\ 29 & 158 \end{array}\right],\;{\text{CM}}_{\text{PHQ}-9}=\left[\begin{array}{cc} 126 & 35\\ 35 & 112 \end{array}\right].$$

Severity-stratified analysis for PHQ-9 (**Table 7**) revealed consistent overall performance, with the highest F1-score in mild cases (0.815) and the lowest in moderate cases (0.698), attributable to greater symptom heterogeneity in the moderate range. High specificity across all strata supports effective rule-out capability for screening applications.

**Table 7:** PHQ-9 severity-stratified classification performance.

Metric	Mild (*n* = 89)	Moderate (*n* = 112)	Severe (*n* = 107)	Overall
Accuracy	0.831	0.723	0.794	0.773
F1-score	0.815	0.698	0.778	0.762
Precision	0.847	0.712	0.789	0.762
Recall	0.787	0.685	0.766	0.762
Specificity	0.892	0.756	0.823	0.823

### 3.4 Training stability and convergence

**Figure 3:**
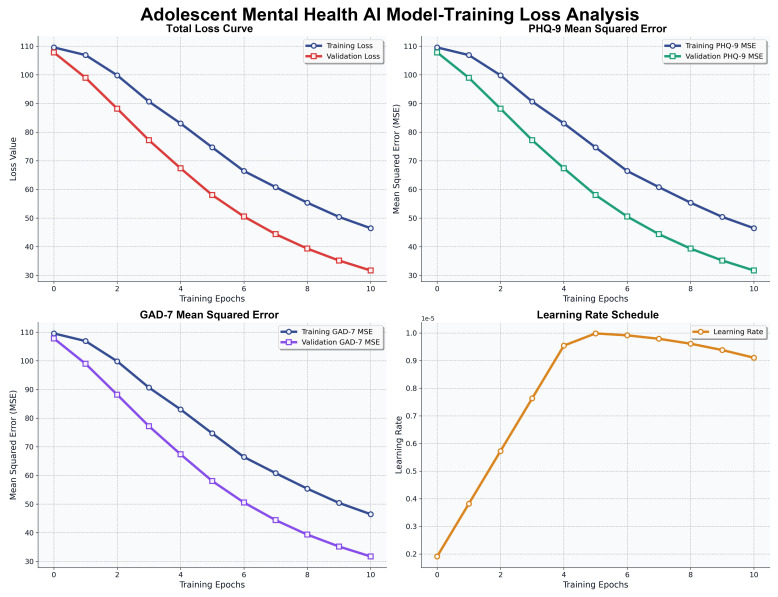
Training and validation loss curves with learning rate schedule.

Training proceeded for 35 epochs with early stopping (patience = 3). As depicted in **Figure 3**, total loss, PHQ-9 MSE, and GAD-7 MSE exhibited monotonic decline, with negligible train-validation gaps after epoch 10. The accompanying learning rate schedule ensured stable optimization. Asymmetric loss weighting prioritized classification while maintaining regression accuracy, confirming robust multitask convergence without overfitting.

### 3.5 Ablation study

Ablation results (**Table 8**) confirmed the contribution of each component. The absence of multitask learning substantially degraded F1-scores (6.2%–7.8%) and increased MSE (14.2%–18.4%). Removing culturally attuned somatization adaptation reduced GAD-7 F1-score by 5.1%, while omitting severe-case augmentation lowered recall in high-risk subgroups by 4.4%–6.3%.

**Table 8:** Ablation study on the validation set (changes relative to the full model).

Configuration	ΔF1-score (PHQ-9/GAD-7)	ΔMSE (PHQ-9/GAD-7)
Full IDS	0 (reference)	0 (reference)
- Multitask learning	−0.078/−0.062	+18.4/+14.2
- Somatization adaptation	−0.051/−0.051	+9.7/+11.3
- Severe-case augmentation	−0.063/−0.044	+7.1/+6.8

## 4 Discussion

The IDS framework developed in this study demonstrates promising diagnostic performance (Pearson *r* ≈ 0.70, AUC *>* 0.87) for non-intrusive screening of depression and anxiety in adolescents. This system provides a comprehensive dual-task assessment specifically optimized for Chinese adolescent populations. These results align with evidence that deep learning-derived digital biomarkers can complement traditional clinical assessments (16). Our correlation coefficients are comparable to those reported for validated Chinese versions of the PHQ-9 and GAD-7 scale in adolescent populations (27, 28). Recent comparative studies further contextualize these findings; for instance, text-based models using social media data for depression detection in Chinese youth have reported AUCs ranging from 0.81 to 0.88 (29), while multimodal approaches incorporating voice and facial cues achieve marginally higher performance (AUC 0.85–0.95) but at the cost of increased intrusiveness (30). Our single-modality, spontaneous-text approach thus offers a favorable trade-off between privacy preservation and diagnostic utility, particularly suitable for large-scale, low-burden deployment in educational settings where routine collection of voice or video data may raise acceptability concerns. The integration of multitask learning with attention mechanisms represents a novel approach that leverages symptom correlations to improve prediction accuracy. Capturing spontaneous linguistic expressions provides a robust “digital phenotype” of adolescent distress, potentially mitigating self-presentation and reporting biases common in manual screening (9). Clinically, this could support school-based triage by enabling early identification of at-risk students without requiring in-person interviews, thereby enhancing intervention efficiency and potentially reducing the overall burden of mental health disorders, with epidemiological data indicating that up to 25%–30% of Chinese adolescents were affected during and after the COVID-19 era (31).

A central innovation of this study is the joint modeling of depression and anxiety, reflecting their high clinical comorbidity in adolescents (rates often ranging from 15% to 75% in clinical samples) (21). Compared to single-task models, our multitask learning framework leverages shared semantic features to improve predictive stability. Furthermore, our asymmetric loss weighting strategy (*β* = 6.0) prioritizes risk categorization. This design choice aligns with clinical utility principles in AI systems, where accurate identification of severe cases is often more critical than minimizing average error in continuous scores (32). In practice, this could optimize resource allocation in school counseling by prioritizing severe cases, although fairness audits are recommended to address potential biases in diverse populations, such as those from rural versus urban areas or different ethnic groups, as highlighted in recent fairness evaluations of AI mental health models (33). Such audits could involve metrics like demographic parity and equalized odds to ensure equitable performance across subgroups, preventing exacerbation of existing disparities in adolescent mental healthcare access in China. The multitask learning architecture successfully captures the intrinsic correlations between depression and anxiety symptoms, achieving enhanced generalization ability through shared feature learning.

The VAE-based data augmentation strategy addresses a critical limitation in mental health research—the scarcity of labeled data due to privacy concerns. To address pervasive class imbalance, we employed a VAE-based data augmentation strategy. Unlike traditional oversampling, this approach learns a continuous latent manifold, enabling the generation of semantically coherent synthetic texts (34). The resulting performance gains in severe cases underscore generative AI’s potential to mitigate data-driven biases against high-risk populations (35). By enriching underrepresented regions of the feature space, the IDS promotes more equitable diagnostic sensitivity. However, synthetic data require further expert validation to ensure clinical appropriateness, as generative methods can introduce risks such as semantic drift or mode collapse, potentially leading to misinterpretations in mental health assessments (35). Specific validation methods could include Turing-style expert reviews or randomized controlled trials comparing model outputs with real patient data. Deployment should comply with privacy regulations such as China’s Personal Information Protection Law, safeguarding against unintended disclosure of sensitive adolescent mental health information.

The Chinese-optimized BERT model demonstrates superior performance in understanding adolescent psychological expressions. The efficacy of our Chinese-optimized BERT encoder underscores the need for localized linguistic analysis in digital mental health tools. Clinical evidence indicates that Chinese individuals, including adolescents, often express psychological distress through somatization—reporting physical symptoms such as fatigue or sleep disturbances rather than direct emotional terms (12). Recent epidemiological studies reinforce this, showing somatization rates of approximately 9%–15% in Chinese adolescent populations, with strong associations to depression and anxiety (13). Leveraging self-attention mechanisms, our model captures these subtle, culturally specific markers, which may be overlooked in generic natural language processing (NLP) tools due to linguistic and cultural biases (33). This approach better aligns with implicit communicative styles in Chinese sociocultural contexts (13). To enhance generalizability, future iterations could account for regional variations, such as differences in urban versus rural expressions or across provinces, where mental health burdens vary geographically (31). Incorporating culturally attuned training, such as integrating traditional Chinese medicine concepts, could further reduce misdiagnosis risks in community or school settings.

The interpretability design represents a crucial advancement for clinical applications, addressing the black-box problem. Despite strong performance, translating the IDS framework to clinical practice requires addressing deep learning’s opacity. While attention mechanisms provide initial insights, integrating *post hoc* techniques such as SHapley Additive exPlanations (SHAP) is essential for human-in-the-loop decision-making and building clinician trust (36). Several limitations warrant careful consideration: the sample size may limit generalizability, the dataset primarily represents urban adolescents, and the text-only approach may miss non-verbal cues. First, the cross-sectional design limits assessment of symptom trajectories; this may lead to overestimation of static risk without capturing fluctuations, underscoring the need for longitudinal approaches crucial for advancing toward prognostic applications (31). Second, although VAE augmentation improved sensitivity for severe cases, synthetic data warrant rigorous clinical validation, potentially through multicenter randomized trials, to avoid propagating biases. Ethical considerations, including potential surveillance misuse in adolescents, necessitate robust privacy guidelines, especially given the vulnerability of this population to stigma and data breaches. Regulatory pathways, such as those under China’s National Medical Products Administration (NMPA) for AI medical devices, should be navigated, involving classification as class III devices and post-market surveillance to ensure safety (32). Finally, multimodal fusion—integrating textual data with acoustic and prosodic features—holds promise for more comprehensive assessments (30), with future work suggesting randomized controlled trials in secondary schools to validate scalability in low-resource environments.

## 5 Conclusion

This study introduces the IDS, a multitask deep learning framework tailored for non-intrusive screening of comorbid depression and anxiety in Chinese adolescents. By incorporating culturally sensitive linguistic modeling and strategies to prioritize severe cases and mitigate data imbalance, the framework demonstrates promising diagnostic performance while addressing key limitations of traditional self-report measures in non-Western populations.

Beyond technical advancements, the IDS highlights the potential of AI to enable scalable, stigma-reducing mental health screening in real-world settings such as schools and community programs—particularly valuable amid rising adolescent mental health challenges in China and globally. However, clinical adoption hinges on continued efforts to enhance model explainability, rigorously validate generative components, ensure ethical deployment, and expand to longitudinal and multimodal paradigms.

Ultimately, tools like IDS should serve as complements to human-centered care. Realizing their transformative promise will require sustained interdisciplinary collaboration to bridge technical innovation with clinical needs, cultural nuance, and equitable access, thereby contributing to a more proactive and inclusive global adolescent mental health ecosystem.

## Data Availability

Publicly available datasets were analyzed in this study. The dataset used in this study is publicly available at: https://github.com/shuyeit/mmpsy-data (Repository: GitHub; Repository Name: shuyeit/mmpsy-data; License: MIT License). The dataset contains anonymized mental health assessment data from 1275 students, including PHQ-9 and GAD-7 scale scores, therapy interview audio recordings, and corresponding text transcripts. All data have been anonymized to remove sensitive identifying information in compliance with privacy protection requirements.
